# *iCollections* methodology: workflow, results and lessons learned

**DOI:** 10.3897/BDJ.5.e21277

**Published:** 2017-09-28

**Authors:** Vladimir Blagoderov, Malcolm Penn, Mike Sadka, Adrian Hine, Stephen Brooks, Darrell J. Siebert, Chris Sleep, Steve Cafferty, Elisa Cane, Geoff Martin, Flavia Toloni, Peter Wing, John Chainey, Liz Duffell, Rob Huxley, Sophie Ledger, Caitlin McLaughlin, Gerardo Mazzetta, Jasmin Perera, Robyn Crowther, Lyndsey Douglas, Joanna Durant, Elisabetta Scialabba, Martin Honey, Blanca Huertas, Theresa Howard, Victoria Carter, Sara Albuquerque, Gordon Paterson, Ian J. Kitching

**Affiliations:** 1 National Museums Scotland, Edinburgh, United Kingdom; 2 Natural History Museum, London, United Kingdom; 3 Science Museum, London, United Kingdom

**Keywords:** Digitisation, georeferencing, sites, collection, database, workflow, museum

## Abstract

The Natural History Museum, London (NHMUK) has embarked on an ambitious programme to digitise its collections. The first phase of this programme was to undertake a series of pilot projects to develop the workflows and infrastructure needed to support mass digitisation of very large scientific collections. This paper presents the results of one of the pilot projects – iCollections. This project digitised all the lepidopteran specimens usually considered as butterflies, 181,545 specimens representing 89 species from the British Isles and Ireland. The data digitised includes, species name, georeferenced location, collector and collection date - the what, where, who and when of specimen data. In addition, a digital image of each specimen was taken. A previous paper explained the way the data were obtained and the background to the collections that made up the project. The present paper describes the technical, logistical, and economic aspects of managing the project.

## Introduction

The Natural History Museum, London (NHMUK) has embarked on an ambitious programme to digitise its entire collections of some 80 million specimens (see http://www.nhm.ac.uk/our-science/our-work/digital-museum.html for background and details). The iCollections project was developed as part of this programme with the aim of developing the necessary data pipelines and digitisation workflows to undertake such a mass digitisation project. In addition to the aim of digitising a large collection, iCollections was also established to test the systems that would have to be developed and the ability of the existing infrastructure to deal with relatively large volumes of data in a timely and secure way. The previous paper (Paterson et al. 2016b) briefly described the resultant dataset and methodology of digitisation. This paper concentrates on details of workflows and the lessons learned, which are fundamental to the design of new digitisation projects (Nelson et al. 2015).

The iCollections project focused on Lepidoptera from the British Isles only; however, for comparative purposes, data from another digitisation project – Crop Wild Relatives, which included several groups of Coleoptera, Diptera and Hemiptera – are included.

Processed specimen records and corresponding images are available on the NHM data portal (http://data.nhm.ac.uk, Paterson et al. 2016a, Paterson et al. 2016b).

## Material and Methods

### Collection of British Isles butterflies and moths

The Rothschild-Cockayne-Kettlewell collection, popularly known as the "RCK", was formed in 1947 from the amalgamation of the Rothschild British and Irish butterfly and moth collection with the extensive combined collections of E.A. Cockayne and H.B.D Kettlewell. Comprising about 2000 drawers of British and Irish butterflies and larger moths arranged to display variation in all its forms, the RCK was originally housed at the Tring Museum but was moved to the Entomology Department of the Natural History Museum (NHM) in South Kensington, London in 1969. The following year, the RCK was merged with the many other important British and Irish Lepidoptera collections already at the NHM to form the present British and Irish Lepidoptera Collection. Now the most important collection of British and Irish Lepidoptera in existence, containing a wealth of material of both scientific and historic importance, the British and Irish Collection comprised, prior to the iCollections project (2013), approximately 500,000 specimens, of which 130,000 were butterflies, housed in 5500 cork-lined drawers and databased to species level only.

All data regarding the number of digitised specimens and associated costs are valid as of January 1, 2016.

### Specimen barcoding

Every specimen was assigned a unique identification number, which was printed using BarTender v. 10.0 in both human- and machine-readable (DataMatrix barcode) formats, as an additional specimen label.

### Imaging

To create specimen stages for digital photography, two standard sizes of specimen unit trays, for smaller and larger specimens, were modified by the addition of a neutral grey background, a scale, and a raised label positioning area on the right side at approximately the same level as the spread wings of the specimens.

Images were captured using Canon EOS 550D and 700D cameras in program or aperture priority mode using Canon EOS Utility software for tethering and image transfer. Custom-built light boxes, each with a 32W Circline VLR Full Spectrum Vita-Lite 5500K fluorescent ring bulb, were used as the light sources.

Image files were stored in a series of folders, named for the drawer locations and taxonomic determination of the specimens (the latter derived from drawer labels).

### Software

Data were captured from the file system structure with a bespoke image processing script that uses ImageMagick-7^®^ (http://www.imagemagick.org) for cropping and Bardecodefiler (http://www.bardecode.com/en1/app/bardecodefiler/) for barcode capture.

Data were stored in a MS SQL Server database. Transcription, data processing and georeferencing tools were provided by a bespoke Microsoft Access 2010 application.

Processed records and corresponding images were ingested into the NHM’s collection management system (CMS), KE EMu (© Axiell), and published through the NHM data portal (http://data.nhm.ac.uk, Paterson et al. 2016b, Paterson et al. 2016a). Flowcharts were prepared using inShort software ver. 1.4.0 (© Yuri Shortki, 2014). The following flowchart conventions are adopted: square blocks - resources; rounded blocks - processes; turquoise blocks - input, red blocks - output, of each workflow module.

### Policies

NHM imaging and georeferencing standards, and data embargo procedures (Suppl. materials 1, 2, 3), were followed. A Transcription Protocol was developed during the initial stages of the project (Suppl. material 4).

## Results

### Workflow (Figure 1)

The selected process is a variant on the “object-to-image-to-data” workflow (Nelson et al. 2012, Nelson et al. 2015). While we deliberately keep descriptions of the various elements general and illustrate the workflow with examples drawn from the NHM collections, other institutions can readily adapt our findings to align with their own internal policies and procedures. At the same time, we concentrate on the general lessons learned from implementation of this type of large-scale digitisation project.

In developing the workflow, we aimed to minimise the amount of specimen handling and other manual operations, and increase division of labour. Complex procedures were divided into smaller, simpler tasks, thereby allowing optimisation of each stage. We found that the most labour intensive and logistically complex stage-imaging and re-housing-was best performed by a pair of digitisers dividing the tasks between them in a sequential-style workflow. Digitisers change roles periodically to reduce monotony.

**Figure 1.**
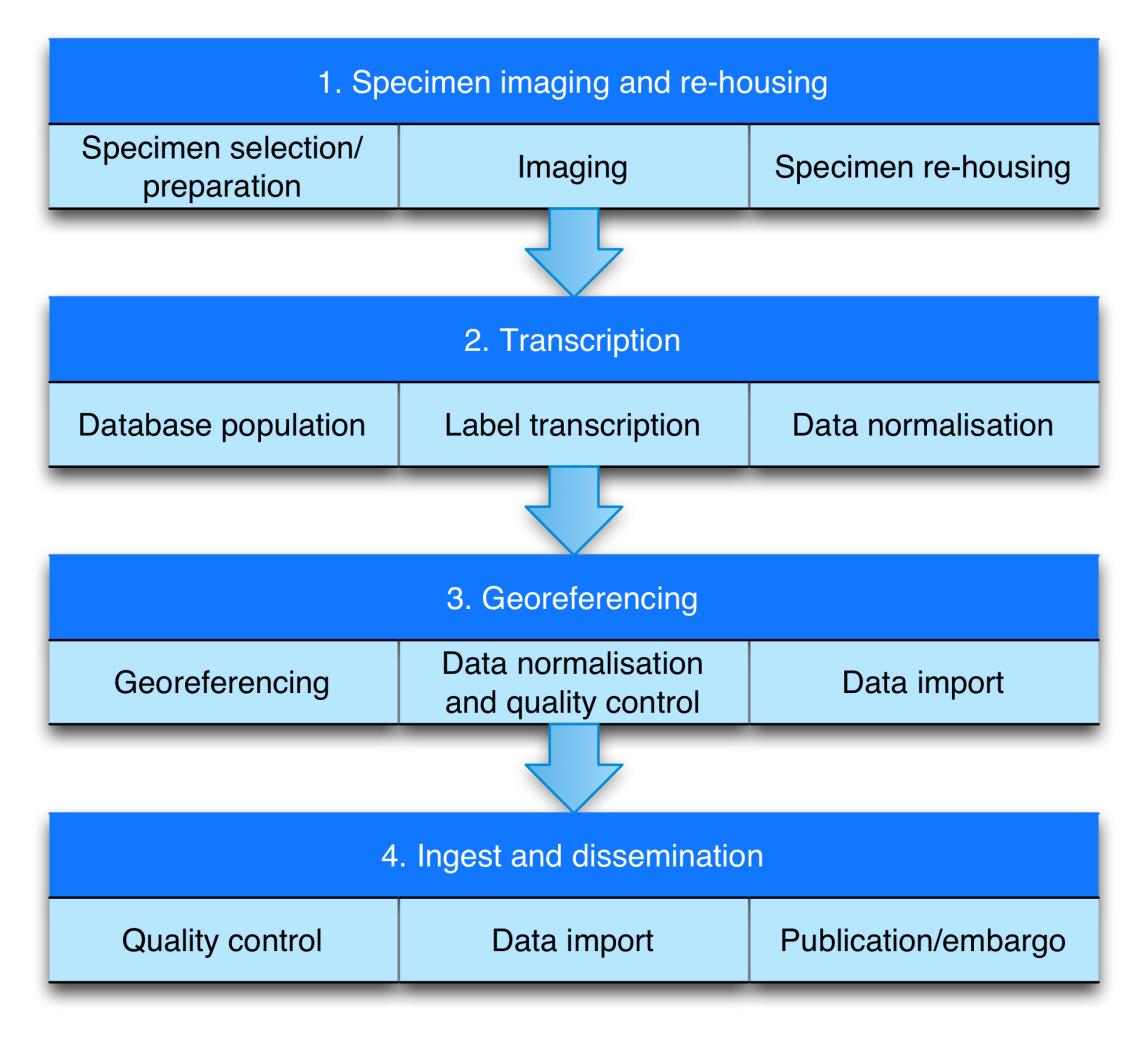
Modular structure of the iCollections digitisation workflow.

The workflow comprises four modules (Fig. 1): 1) Imaging and re-housing; 2) Transcription; 3) Georeferencing; and 4) Ingest into CMS. The modules are independent and can be performed sequentially or in parallel. Towards the end of workflow, the role of data managers increases and that of digitisers and curators decreases. Details of the workflows comprising each of the modules are given in the following sections.

#### Module 1: Specimen imaging and re-housing

**Figure 2.**
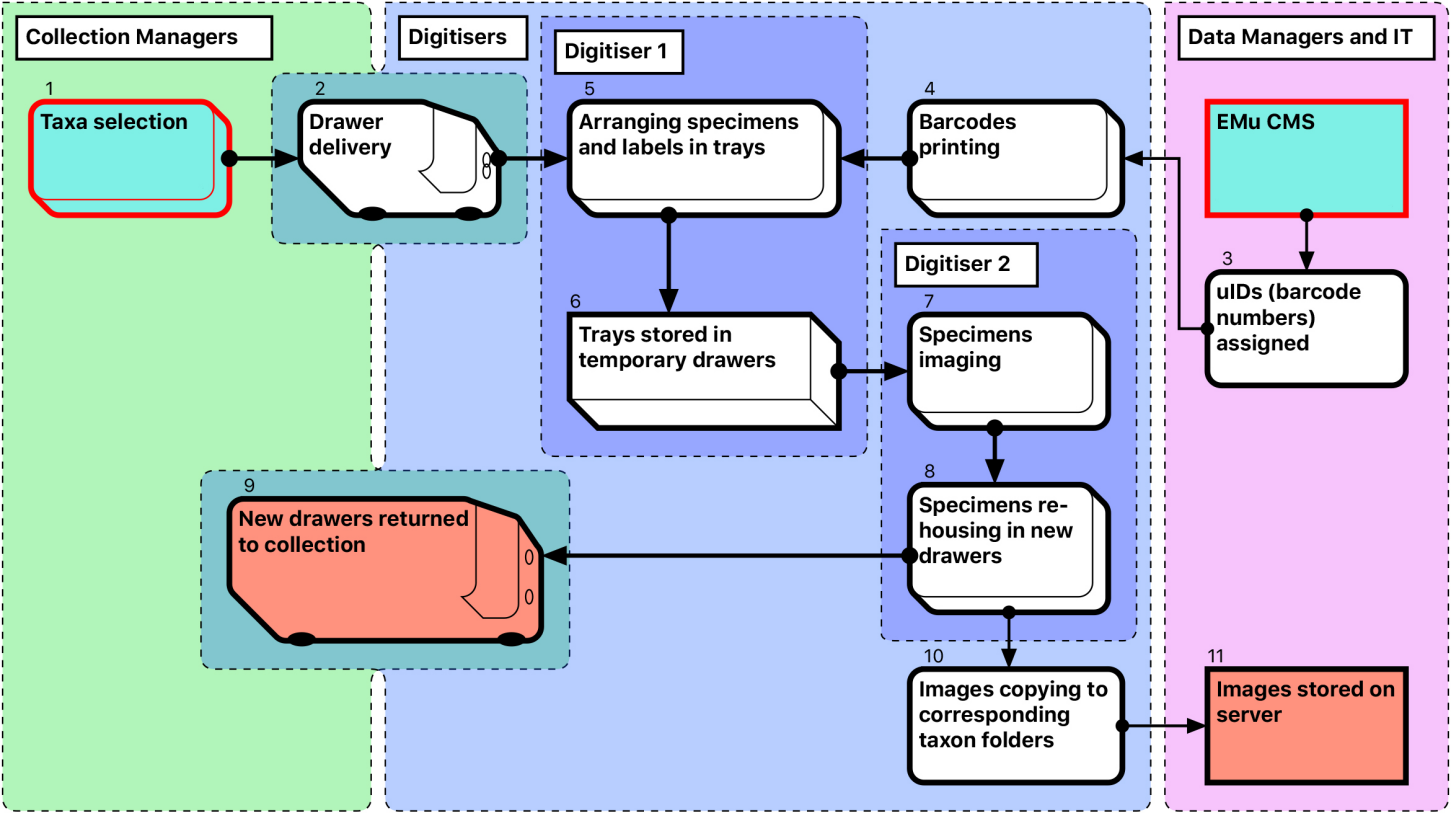
Module 1 (Imaging and re-housing workflow).

The steps in the Module 1 workflow are as follows (Fig. 2):

1. Curators prioritise taxa to be digitised, and prepare and collate all relevant specimens, which are often stored in different locations. A taxon-based approach to digitisation simplifies the process for digitisers who may not be familiar with the organisms.
2. Drawers are delivered to digitisation stations (by curators and/or digitisers) and stored there temporarily while being digitised.
3. Data managers allocate unique numbers to be used as barcodes.
4. Barcode labels are printed at an appropriate size, depending on the size of specimens being digitised. If labels are misplaced, the corresponding barcode numbers are discarded. Steps 3 and 4 together provide uniqueness of object barcodes.
5. The first (preparator) digitiser takes a specimen from its drawer, removes its labels and pins it into a unit tray of appropriate size. The labels are placed on the raised section on the right side of the tray next to specimen. If the specimen does not have a unique ID, a barcode label is added. For specimens that have already been databased, barcode labels are not printed (approximately 1% of specimens).
6. Sixteen specimens are prepared in this way at one time and arranged in a temporary storage drawer prior to imaging.
7. The second (imaging) digitiser takes the prepared drawers, removes a unit tray for imaging, and photographs the specimen and labels. Double-sided labels are photographed from both sides, resulting in two label images per specimen. Images are stored initially (and temporarily) on the local computer HDD.
8. Labels are then reattached to the specimen, which is pinned into a new, permanent drawer. The remaining 15 specimens in the temporary drawer are imaged in the same way. This process is repeated until either the new drawer is filled or the taxon unit is completed. Preparation of specimens takes approximately the same time as imaging and re-housing (see Discussion: Digitisation performance).
9. The new drawers with digitised specimens are returned to the collection space.
10. For each specimen drawer, a folder is created on the server, with subfolders reflecting its taxonomic content. Images are then moved from the local computer HDD to the appropriate folder and subfolders.
11. When each specimen lot (taxonomic unit or drawer) is completed, the imaging digitiser moves the acquired images to a designated server location, maintaining the folder structure (Drawer#/Taxonomic name).

Compromises were necessary at this and subsequent stages to optimise overall productivity (see Discussion). The total number of imaged specimens and numbers of images are shown in Table 1.

#### Module 2: Transcription

**Figure 3.**
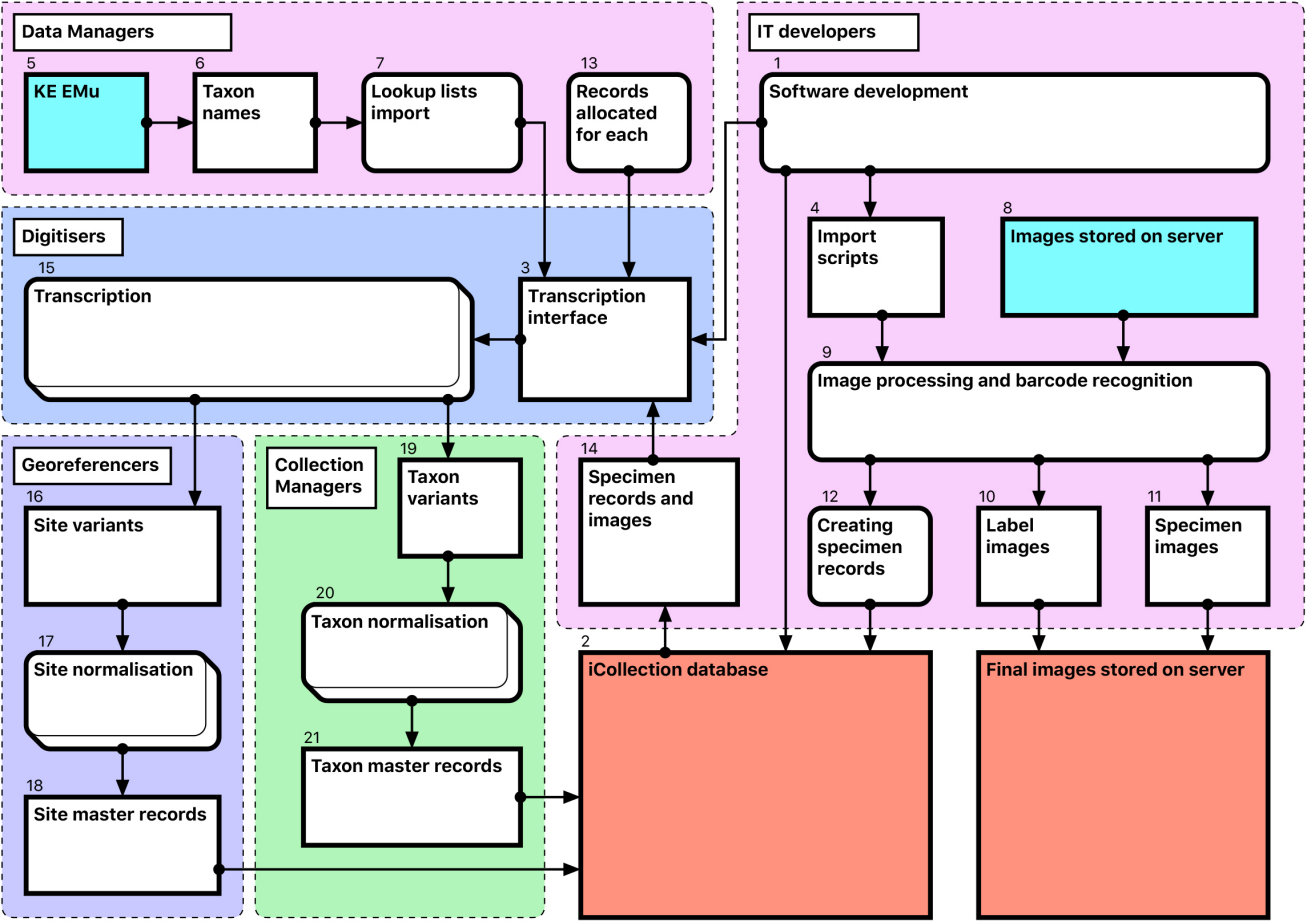
Module 2 (data transcription and normalisation workflow). Bracketed numbers in the following text reference the workflow steps in this diagram.

Module 2 consists of several consecutive steps (Fig. 3):

**Software development [1-4**]

The NHM’s KE EMu (© Axiell) collections management system is unsuited for rapid data entry and includes few tools for data processing. Therefore, we developed a separate Microsoft SQL Server database (the “iCollections database” [2]) to support temporary storage and processing of transcribed data. A graphical user interface (the “iCollections interface” [3]) was developed using MS Access 2010, and provided data transcription, management and processing tools, as well as user administration and reporting capabilities. Considerable effort was invested in optimisation of the user forms to ensure that they were intuitive to use and supported a natural flow of activities.

Scripts [4] were developed to process images, extract metadata from the folder structure, read barcodes from photographs, and import image metadata into the iCollections database.

**Database population [5-12**]

A list of accepted UK lepidopteran taxon names [6] was downloaded from EMu [5] and used to seed the iCollections database lookup taxon list [7]. This is not required but saves time later in the process. Site and collector data could also have been seeded had appropriate data been available.

Users were added to the database, and assigned one or more roles (e.g., transcriber, georeferencer). The system uses these roles to control user access to application functionality.

Scheduled scripts traverse the image storage folders [8] overnight and perform the following operations for each image [9]:

1. Crop the right 25% of image showing the specimen’s labels, resize it to 800 by 1219 pixels and save it as a new file with the original filename suffixed with “_label”.
2. Read the embedded barcode from the new label image.
3. Rename original specimen and label images using the barcode as filename and retaining the “_label” suffix (if barcode recognition is unsuccessful, the original file name is retained).
4. Create a specimen record in the iCollections database, including the taxon name and drawer location (read from the folder path) and the specimen barcode.
5. Create image records, including the image path and filename, associated with their corresponding specimen records.

This produces a record for each specimen, typically linked to 2 image records – the full specimen image and the labels crop [10-12]. This process is scheduled, so once images are deposited on the server, corresponding records are automatically generated in the iCollections database.

**Transcription [13-15], Fig. 4a**

Once the database is populated, a data manager can allocate specimen records to available transcribers [13]. When a transcriber logs into the application they only have access to those records allocated to them. The data manager may also reallocate records if necessary (e.g., to optimise transcriber effort or cover absent staff). This pre-allocation of records simplifies aspects of workflow management, and increases the likelihood that digitisers will work on sequential specimens from one site, helping to minimise interpretation errors.

The transcription interface displays the label crop (if necessary the digitiser can view the corresponding specimen image) and fields for entering or selecting data values [14-15]. Considerable effort was expended to ensure that the interfaces were simple and intuitive to use, supported a natural flow of activities and presented unambiguous choices wherever possible.

Transcribers can access records they have worked on previously, which is vital in allowing them to compare labels or to correct transcriptions if new labels indicate a previous error. This facility to return to and correct previously transcribed records also reduces the pressure on transcribers to “get it right first time”.

Transcribers were asked to capture various data elements from the labels, primarily dates, site and collectors. They were able to indicate uncertainty for some data elements, allowing for later scrutiny and resolution by a subject matter expert (usually a curator). Records with technical issues can be referred to a data administrator. Transcribers are also able to correct barcode misreads (which the application indicates) manually, forcing renaming of the associated image files, and maintaining the correspondence between barcode and filename.

The transcription process was designed specifically to capture verbatim data, without interpretation. As new sites or collectors were encountered on labels, transcribers were able to add them to the database, after which they were visible to all other transcribers and selectable from drop-down menus, reducing the need to key data and the risk of transcription errors.

This separation of transcription and interpretation has two significant implications for site data:

1. A digital version of the verbatim label data is recorded for each specimen.
2. Numerous different renderings (or “variants”) of the same physical site are captured (e.g., “Reading”, “Reading, Berks”, “Redding”), requiring a subsequent normalisation process to remove logical duplicates and standardise the records.

**Data normalisation [16-21], Fig. 4b, c, d**

Both taxon and site “variants” (different literal transcriptions of the same logical concept) are processed by subject matter experts (curators/taxonomists or georeferencers) via the iCollections application. Each variant is assigned to a separate “master” record, which is a standardised version of the concept represented by the variant. The interfaces show the list of variants to the left and allow a user to associate them with a master from the right-hand list. Users may also create new master records as appropriate.

For example, taxon variants “*Aus bus*” and “*A. bus*” might both be associated with the single taxon master record “*Aus bus* (Linnaeus 1758)” [19-21].

Similarly, “Reading”, “Reading, Berks” and “Redding, Berks” variants would all be associated with a single “Reading, Berkshire, UK, Europe” master record. This significantly reduces the georeferencing burden – the reduced set of masters effectively covers all the associated specimens (Table 2) [16-18].

When complete, normalisation ensures that all specimens from a single location or taxon are linked to the same, standardised representation of that location or taxon concept, effectively deduplicating the raw variants [18, 21].

Note that variants and masters are, logically and physically, separate database entities. Variants represent verbatim and/or historical site or taxon strings, each stored just once to ensure transcribers are not required to rekey the same values repeatedly.

The normalisation processes can run in parallel with transcription, with subject matter experts normalising sites and taxa as they are generated by the transcription process.

The site normalisation interface includes a georeferencing tool, so the normalisation and georeferencing processes are typically, although not necessarily, performed simultaneously by one user (Fig. 4d).

**Scrutiny**

Users with the “scrutineer” role have access to the raw data for quality assurance purposes. They can filter and sort specimen records in a traditional data grid, then view and edit the full details of any individual specimen.

This allows data administrators or subject matter experts to more easily identify, assess and remedy data quality issues, including accessing those records that digitisers have flagged as needing expert attention.

Scrutiny was not a formal step in the workflow, but was performed as required at any stage.

#### Module 3: Georeferencing

The iCollections application includes a simple georeferencing tool that allows a user to acquire point and extent data from Google Maps (Fig. 4d). We follow the NHM Georeferencing standards (Suppl. material 2), which give specific and repeatable methods of georeferencing features or places. These can include precise localities such as 4km along the A9 from Perth, nearest named place or the mouth of a river. By following the NHM guidelines we have standards that can be replicated and are based on best practice and are freely available at the NHM.

**Figure 5.**
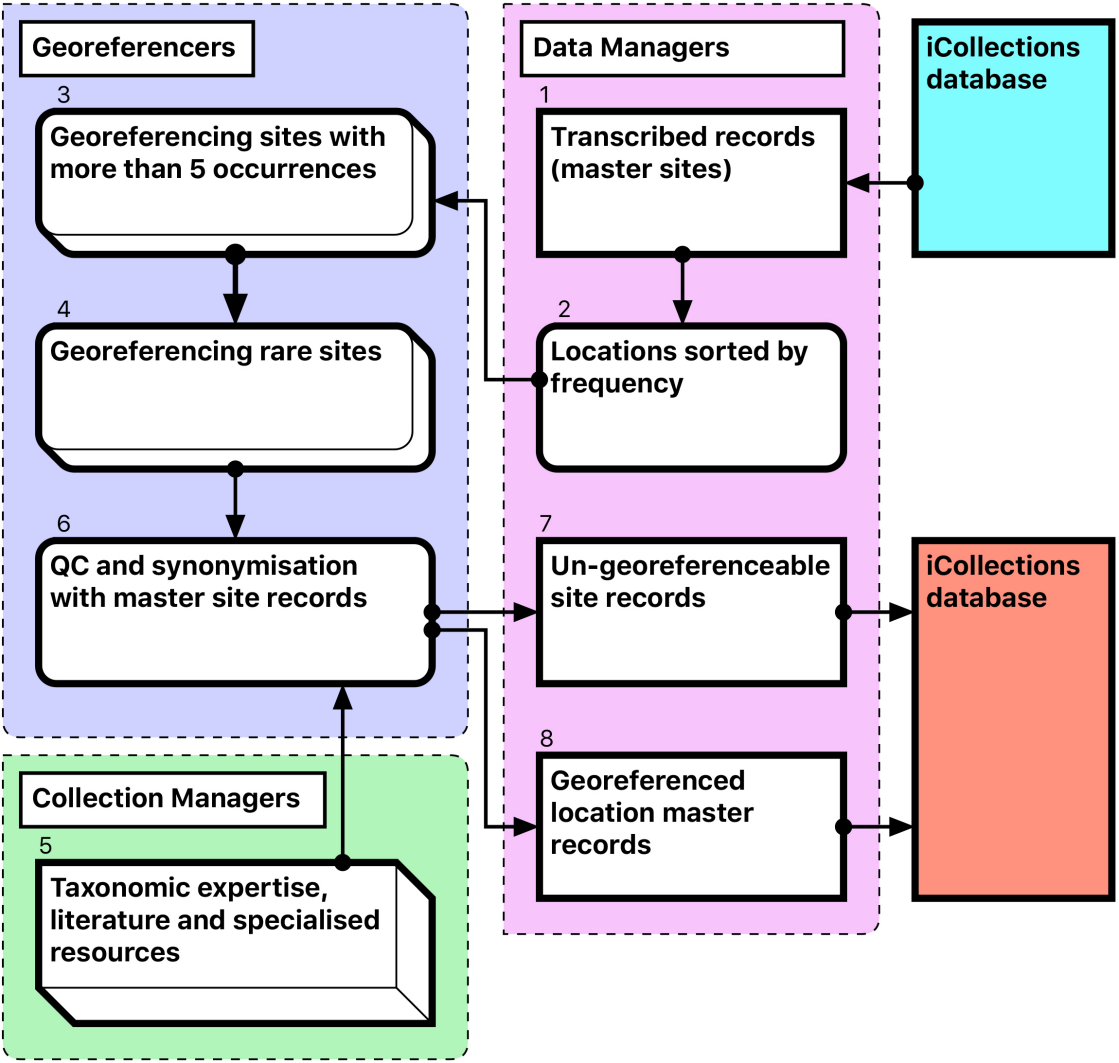
Module 3 (georeferencing workflow). Bracketed numbers in the following text reference the workflow steps in this diagram.

The Georeferencing process is split into five broad stages (Fig. 5).

1. During this stage we only georeference sites with at least five collected specimens. This enables sites with many specimens to be georeferenced quickly, which can account for 60-70% of each collection [1-3].
2. The team splits the remaining data into two parts A-M and N –Z, and each georeferencer spends no more than 15 minutes on each site attempting to georeference the data [4].
3. The remaining data are checked by the team leader and questions answered if possible. The data are then investigated further by the georeferencers and the specific curator /researcher, who provide help on any specific problematic locations [5].
4. The remaining data are checked by the team. Sites that cannot be georeferenced are noted as “un-georeferenceable” in the data [6-7]. The accuracy of the data set is checked by selecting 100 sites at random from the data and comparing the results of two independent georeferencers.
5. The data are then exported and ingested into KeEMU by Database team [8].

#### Module 4: Ingest and dissemination

**Figure 6.**
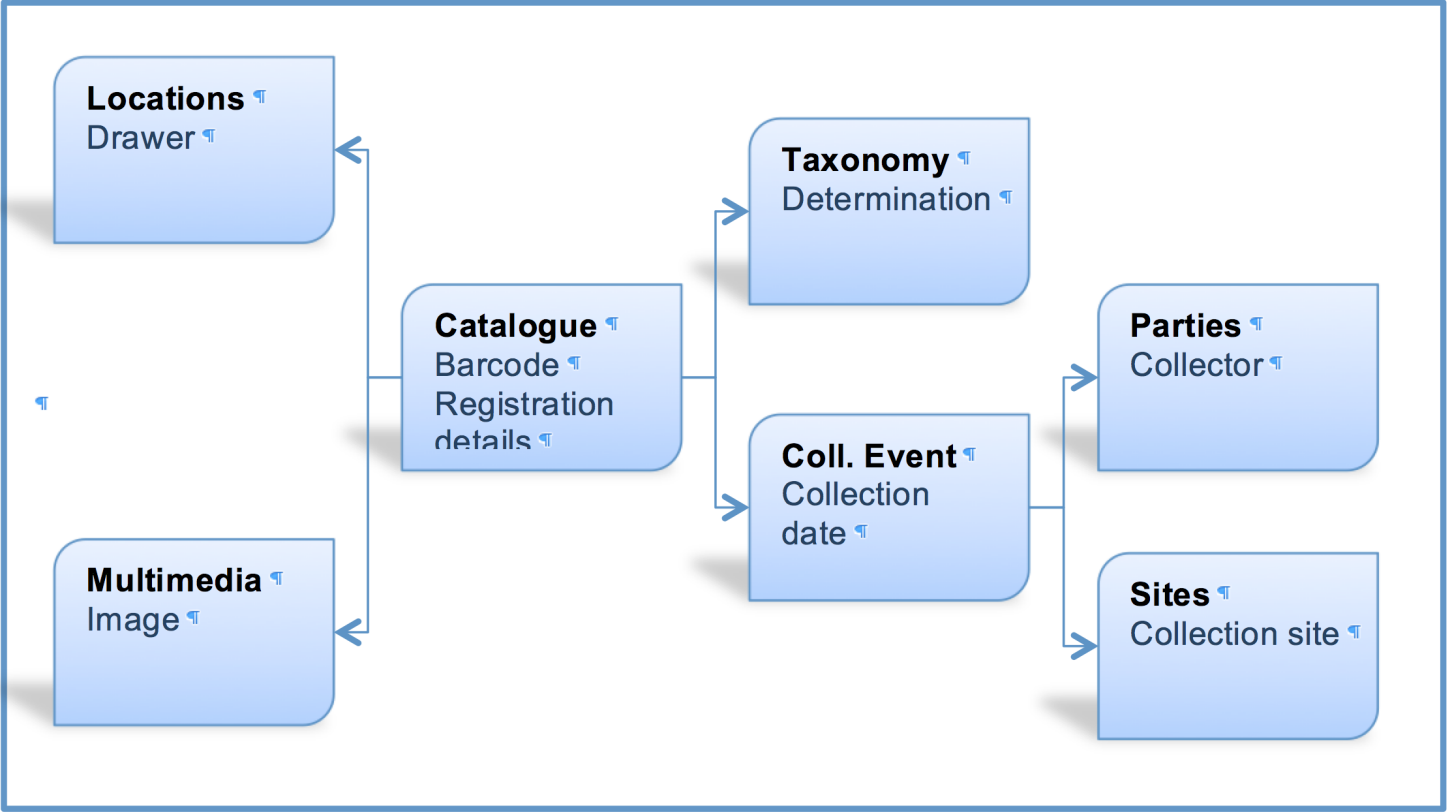
Logical relationships between relevant data types in KE EMu.

EMu records reference other records within the system via IRNs (Internal Record Numbers – unique record identifiers) according to the schema shown in Fig. 6. Records must exist before being referenced, so the order in which entities are imported is critical. Where possible, pre-existing CMS records are referenced (to avoid duplication). So iCollections records are compared with those already in EMu, and any IRNs for pre-existing records are recorded in iCollections, allowing new records from iCollections to reference pre-existing EMu records.

**Figure 7.**
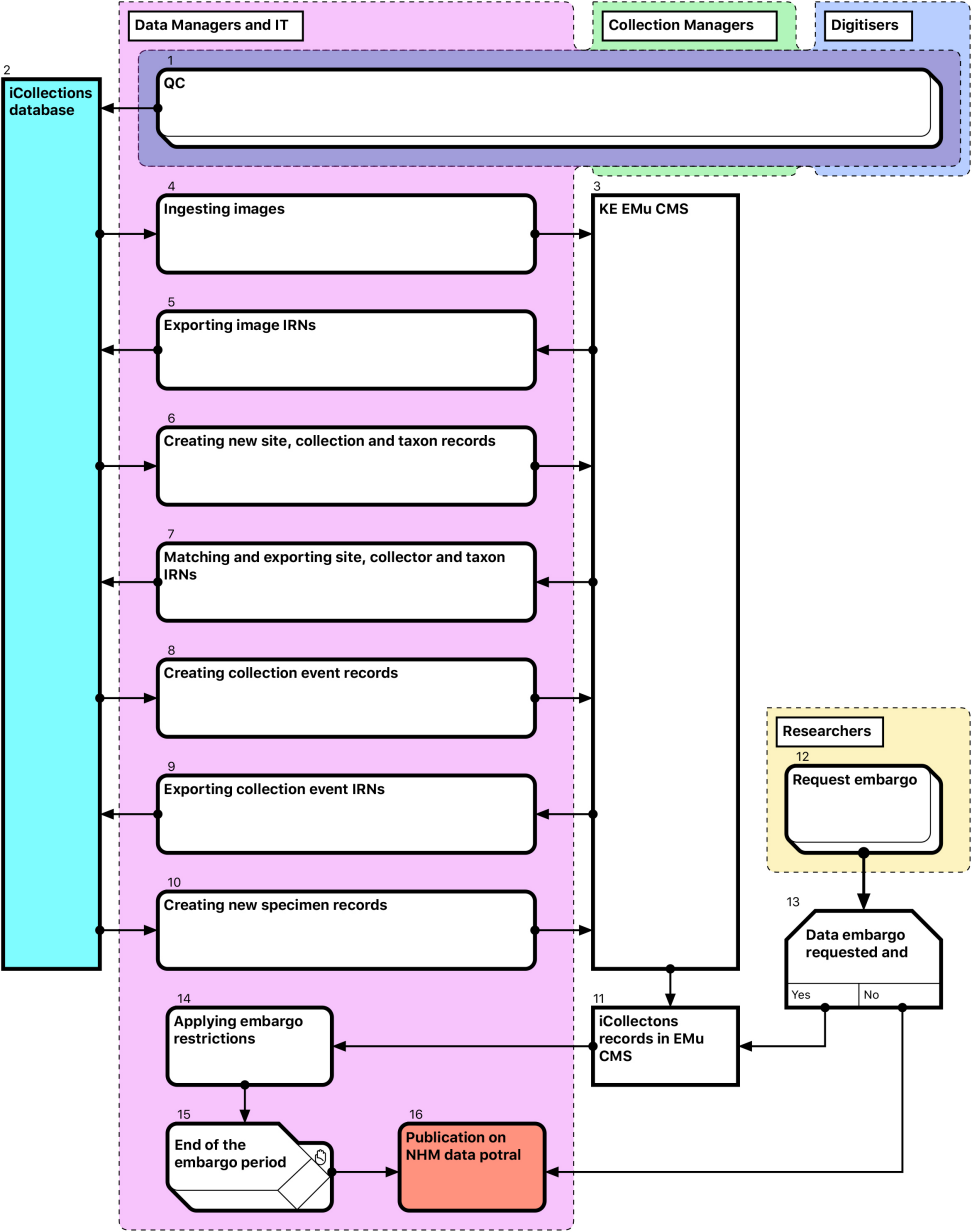
Module 4 (Ingest and dissemination workflow). Bracketed numbers in the following text reference the workflow steps in this diagram.

Thus, for each referenceable record type (image, collector, site, collection event or taxon) the following steps are required (Fig. 7):

1. Create new EMu records of an appropriate type, if not already present.
2. Retrieve matching EMu IRNs and store with corresponding iCollections records (for later use as a reference).

After Quality Control (QC) [1], transfer of data from iCollections [2] into EMu [3] is a multi-stage process, with new entity records being ingested into EMu and EMu IRNs (except those for specimens) returned to iCollections [4-10]. This process is mediated primarily by generation of csv reports containing new records from iCollections and corresponding IRNs from EMu.

Data embargo procedures are then applied as appropriate [12-15] before both images and specimen data are released through the NHM Data Portal (data.nhm.ac.uk) [16].

Once Data Managers are assured that specimen records have been ingested correctly, they have no further utility in the iCollections database and may be archived.

### Collection results

Post-digitisation, the British and Irish Lepidoptera Collection is now housed in plastazote-lined drawers, with all outlying collections amalgamated and including a considerable number of specimens extracted from the world collection. The complete collection comprises 181,545 specimens housed in 1360 drawers. All specimens have been individually imaged, databased and georeferenced, and are now available for study through the NHM Data Portal and GBIF. Every UK butterfly specimen in the NHM has now been collated into this updated British and Irish Lepidoptera Collection and consequently the collection has ‘grown’ from a previous estimate of 130,000 specimens to an actual figure of 181,545 (Table 3), an approximately 40% increase. The Macro-moths digitisation is currently about 50% complete (as of October 2016), with 267,318 specimens digitised and housed in 1457 drawers. These specimens will also be made available through the NHM Data Portal in due course. This work has virtually eliminated the need for specimen handling by researchers and visitors and will greatly speed up our response time to collection enquiries.

### Digitisation infrastructure

One of the most obvious achievements of the iCollections project was the creation of a digitisation infrastructure at the NHM, thereby maintaining a high public profile for digitisation as an activity:

- A team of experienced digitisers was assembled.
- A dedicated digitisation operations zone was established.
- Infrastructure to capture, store and handle images and data was developed, including robust and adaptable user interfaces to capture, normalise and georeference label data.
- The digitisation group was featured in events such as Science Uncovered (part of European Researchers’ Night), Nature Live and the Digital Horizons meeting, and has hosted at least 40 visiting groups from government, business and other museums.

### Software

In its current form, the iCollections application provides the following tools:

- Label data transcription (potentially from any label image).
- Taxon record normalisation.
- Site record normalisation.
- Georeferencing.
- Data scrutiny.
  - The specimen dataset may be examined, filtered and sorted in a split screen, which also allows the selected record to be viewed in detail and edited if necessary.
- Administration (Fig. 8).
  - Project configuration.
  - User workload management.
  - User activity reporting.

In principle, the iCollections client application is suitable for transcription of any kind of label image including, for example, herbarium sheets and microscope slides. For some collections, additional fields might be needed (e.g., to accommodate stratigraphy), which would entail further development.

Multiple projects can occupy a single instance of the database and be managed via the same front end application. Projects may be configured independently, including (inter alia) what data to capture and whether or not to utilise taxon/site variants/masters from other projects.

Users can be assigned roles in one or more projects, determining the data and tools they may access.

### Research output

The digitised collection has proved an invaluable resource for research into the responses of British butterflies to long-term climate change. (Brooks et al. 2014) have shown that, in each year, the 10th, 50th and 90th percentile collection dates reflect the flight period of each species, and that 10th and 50th percentile collection dates advance in years with warm springs and summers, and are delayed in years with cool and wet springs and summers. By using the digitised images of the Silver-spotted Skipper, *Hesperia comma*, to make accurate measurements of wing length, (Fenberg et al. 2016) were able to show that males become larger with increasing June temperature. The species’ first date of flying is more advanced in years with high July temperatures and later when July is cool, and the distributional range of the species in southern England expands when August temperatures are warm, but contracts when August is cool. The caterpillar is in its final instar during June so is able to feed for longer and grow more when temperatures are higher, leading to larger adult insects. The species is in its pupal stage during July and so July temperatures influence the emergence date of the adult. August is the main flight period of the adult butterfly and it will disperse further and establish new colonies when that month is warm.

## Discussion

### Comments on decisions made

Several compromises had to be made to optimise the performance of various stages of the workflow.

#### Imaging and re-housing

- Ideally, physical curation of the collection should be undertaken as a preliminary step before digitisation. However, in the present implementation, the collection had to be moved from old drawers with a cork lining to new, plastazote-lined drawers. Thus, to minimise specimen handling and potential damage arising therefrom, collection re-housing was combined with imaging.
- The most effective way to image specimens within the project was for digitisers to work in pairs, rather than individually or as part of a ‘production line’. This proved to be a flexible means of tackling the digitisation of butterfly specimens. Each pair worked on a separate species. If species were represented by more than one subspecies, then these were sorted prior to imaging, which increased work rates. The digitiser pairs collaborated to solve problems as they arose and to develop effective solutions.
- The use of in-camera high quality JPEG images instead of uncompressed file formats reduced post-processing time, file number and disc storage requirements. Image quality was determined as sufficient for research and curatorial purposes and this format needed no further editing for display on-line. The original master images will be stored in a digital asset management system (DAMS) that prohibits unauthorised editing, so the risk of unintended compression (should a user re-save a JPEG) is negligible.
- The use of standardised unit trays saved time when framing and focusing.
- Image consistency was ensured by use of identical light boxes and a standard white balance setup.
- Temporal separation of imaging and transcription made import and data checks easier for data managers and curators. Periods of three weeks’ imaging were followed by approximately one week of transcription that involved all digitisers. Georeferencing, a completely independent stage in the workflow, typically started much later to allow accumulation of sufficient transcribed records for frequency analysis.

#### Automation of data capture

- Wherever possible, image processing and data capture were automated so as to populate the database rapidly prior to transcription, and to facilitate transfer of images and metadata to KE EMu. This minimised impact on the transcription process (by not diverting digitiser effort) and reduced the potential for errors.
- Extraction, down-scaling and sharpening of the label area of the specimen image increased speed and accuracy of barcode recognition.
- Cropping of the label area, barcode recognition and file renaming was scheduled as an overnight batch process. The database was pre-populated with drawer, label, barcode and taxon data using a script that navigated and read the folder structure of the file system: Drawer ##/Taxonomic name/Barcode ##.jpg.

#### Transcription, normalisation and import into KE EMu

Transcription aimed to capture data for both curatorial and research purposes. However, it proved impractical to transcribe absolutely everything on the labels (due to, for example, cryptic conventions, abbreviations and/or non-standard characters/symbols on labels), so transcription focused on a restricted number of core data components (Table 4).

The taxonomy normalisation step is necessary to remove erroneous taxonomic names (e.g., due to mistyping at the imaging stage or incorrect drawer labelling) (Fig. 4b). Such names are linked by curators or collections managers (as taxonomic experts) to the appropriate name in the master list (new master list entries being created as necessary). Likewise, the site normalisation step is necessary to reduce the number of sites that have to be georeferenced. Both of these processes facilitate the eventual import of the data from the iCollections database into KE EMu, with the minimum conflict with pre-existing data types (Fig. 7). Existing records in KE EMu should be used where available and possible, but for some data components new KE EMu records have to be created and other adjustments made (Table 5).

When importing records from iCollections to EMu it was important to avoid creating duplicates, so iCollections records needed to be either (a) matched to an existing record of the same concept in EMu, or (b) imported into EMu as new records. The action(s) taken for each entity are listed in Table 5.

#### Software

MS Access was chosen primarily for its speed and the ease with which comprehensive and functional data interfaces can be created. Its flexibility and compatibility with existing NHM IT systems and skillsets were also significant factors.

#### Data Quality

Quality control and assurance (QC/QA) procedures are implemented at all stages of digitisation so tackling data quality is a multifaceted task involving different teams and expertise (digitisers, data managers, curators and georeferencers). Errors may occur at any stage of the digitisation process or be present in the original label data. Errors are inevitable and so a strategy is required to minimise, catch, categorise and resolve them. QC/QA procedures aim to ensure correctness, completeness, and consistency of data.

Principal sources of errors:

- Digitisation process errors:
  - Imaging (upside down, blurred, multiple, wrongly associated, missing).
  - Barcode (misread, no barcode, two barcodes).
  - Wrong taxon.
  - Wrong drawer.
  - Non-British specimen.
- Transcription errors:
  - Unreadable labels (poor handwriting).
  - Misinterpreted data (‘coll’ => collector or collection, incorrectly assumed century).
  - Missing information (information accidentally skipped).
  - Errors on the data labels:
    - Conflicting localities.
    - Wrong dates.
    - Multiple/erroneous registration numbers .

The principle QC/QA measures taken were:

- Normalised database structures with appropriate indexing and data validation rules.
- Visual highlighting of specific issues (e.g., barcode read failures) in the transcription interface.
- Automated scripting for file management (renaming, cropping and copying) and data import/export tasks.
- Option for transcribers to flag unclear records for escalation, either ‘for scrutiny’ (curator) or ‘refer to admin’ (data manager), and resolution.
- Data checks in the backend databases at different stages, both pre- and post-import.
- Documented data capture protocols, training and support.
- Vigilance and diligence of digitisers, data managers and subject matter experts.

### Managing the project

The principle management activities were identified during the planning stage and a series of work packages (WP) established. Each WP had a designated lead person responsible for ensuring delivery of the WP’s objectives. The WPs were developed around a basic workflow. Clear description of all processes and separation of independent steps in the workflow allowed us to use elements of project management methodologies, such as PRINCE2 (OGC (Office of Government Commerce) 2009) and Critical Chain Project Management (Goldratt 1997). Application of the principles of project management, such as proper scheduling, removal of unnecessary multitasking, and understanding of resource dependencies in the project, allowed us to prioritise and optimise iteratively each stage of the workflow.

The initial phase of the project focused on optimising the workflow. This pilot phase enabled us to identify that, of all the elements, the imaging process took the longest time and its optimisation had to be prioritised. Working in teams and using unit trays for temporary storage of the specimens decreased the time for imaging and rehousing from almost five minutes to 1.85 minutes per specimen (Table 6). Next, the transcription stage was optimised, primarily through development of a user-friendly interface, which lowered the average time per specimen from 0.84 minutes to 0.55 minutes per specimen.

For day-to-day project management, a simplified version of the management methodology characterised as “Common sense, Open communication and Good judgement” (COG) was used. The Chair of the project (GP) combined the functions of SRO and Project Manager; work package leaders were responsible for delivering corresponding streams of work. Weekly meetings of the entire team and other staff engaged at particular stages ensured transparent communication and decision-making.

#### Digitisation performance

To provide a baseline for digitiser performance in similar future projects of pinned insects, we timed the tasks performed by individuals throughout the project. The data were analysed and compared with timings measured for similar projects (e.g., the Diptera Digitisation Project). Base performance (*E*) was estimated using a three-point method with following parameters: *a*, the minimum time as a best-case estimate; *b*, the top 90% of maximum time, a worst-case estimate; and *m*, the median, 50%, chance of completion, a most likely estimate. Performance is then calculated using the formula:

\begin{varwidth}{50in}
        \begin{equation*}
            E = (a+4m+b) / 6
        \end{equation*}
    \end{varwidth}

with the standard deviation estimated as

\begin{varwidth}{50in}
        \begin{equation*}
            SD=(b-a) / 6
        \end{equation*}
    \end{varwidth}

which is widely accepted in management and information systems as producing good project estimates based on limited information. Digitisation time per specimen varies greatly and depends on taxonomic and geographic breadth of the collection and its curatorial quality. Generally, British butterflies and moths were collected in relatively few localities (<5,000), so transcription and interpretation of labels was easier and faster (37.6 specimens per site in average) than for the Diptera collection, which had a similar number of sites, but worldwide, and fewer specimens (8.4) per site. Handling smaller specimens also takes longer (Table 6). Georeferencing times cannot be calculated directly based on number of specimens, but only estimated based on sites due to the nature of georeferencing process. Approximately 300-350 site variants can be dealt with by one person per week.

#### Project costs

In total, 13 people were employed as digitisers for a total duration of 264 months, of which 197 months were spent on the iCollections project (Table 7). Additional direct costs included equipment and consumables, and refurbishment of the digitisation suite. However, the total cost of the project to the NHM is much higher, as that also includes the salary costs of all involved personnel: curators, researchers, IT specialists, data managers, facility managers and administrative staff. These costs are particularly significant during the initial planning and set-up stages of the project, but may be partly reduced in future projects once workflows and protocols have become established (for example, compare the costs of the earlier Butterflies and the later Moths subprojects in Table 7).

In assessing the economics of the iCollections project, we have included all relevant costs, particularly those relating to institutional infrastructure, as we consider that these staff and their associated costs are often overlooked by other digitisation projects. These are true costs, which, while not always acknowledged in funding applications, do nevertheless support critical activities. For example, interface development, together with data transfer and storage, is heavily reliant on institutional infrastructure and access to relevant personnel. In developing iCollections we found that the project required interaction with many different areas of the NHM: human resources, administration, curation and research. As with digitisation performance, costs are also incurred depending upon the nature and curatorial quality of the collection. The better curated the collection, the fewer extra staff will need to be involved in the project and thus the less expensive will be its digitisation. The easier specimens are to handle and the narrower the scope of the project (taxonomic and/or geographical), the less time will be spent digitising each specimen.

### Lessons learned

Collections:

- Curatorial support is crucial at all stages for delivery and return of specimens, labelling, label disambiguation, data scrutiny, general enquiries, and identification of unsorted material.
- Collection space expansion may be necessary.

Data workflow:

- The amount of data management should not be underestimated; it underpins every stage of a digitisation workflow - planning, data capture, enhancement and normalisation, and import into a CMS.
- There is a tendency to think “digitisation = transcription” and that is the job done; it most certainly is not. Transcription is only the start of the process to produce data fit for purpose.
- Factor in a significant time contingency for the large number of things that will go wrong. Some can be anticipated, but others will emerge from the process itself. Be prepared to be reactive.
- There are many facets to data quality and these have to be managed throughout the process. Do not underestimate the training, support and documentation required.
- Consider any pre-existing data – how will you treat existing specimen records? In iCollections, existing specimen records were simply deleted.

Data automation:

- Automation scripts should be modular, for easier management and reuse.
- Avoid hard-coded configuration parameters.
- Optimise from the outset. Capacity and performance needs will grow as capture proceeds.
- Ideally data should be captured directly into the collections management system.

User interface:

- Work closely with users; small interface conveniences can achieve significant efficiency and user satisfaction gains.
- Design and layout are not superficial - subtle changes can significantly improve usability and therefore productivity.
- High data quality is easier to achieve prior to import into a collections management system like KE EMu.
- Varied project requirements can be managed in a relatively simple system.

Georeferencing:

- Standards for locality information can vary significantly across collections.
- Curator / Researcher interaction is necessary to supplement georeferencing expertise with expert knowledge.
- Semi-automated georeferencing functions based on Google Maps and various georeferencing software tools, such as Biogeomancer, allow at least 10% of the total sites variants to be georeferenced quickly and accurately.

## Supplementary Material

Supplementary material 1 NHM Imaging Standards Data type: PDF
File: oo_146751.pdf Vladimir Blagoderov

Supplementary material 2 NMH Georeferencing Guidelines Data type: PDF
File: oo_146752.pdf Malcolm Penn

Supplementary material 3 NHM Data Embargo Policy Data type: PDF
File: oo_146753.pdf Matt Woodburn

Supplementary material 4 iCollections Transcription Protocol Data type: PDF
File: oo_146754.pdf Geoff Martin, Flavia Toloni, Peter Wing

## Figures and Tables

**Figure 4a.**
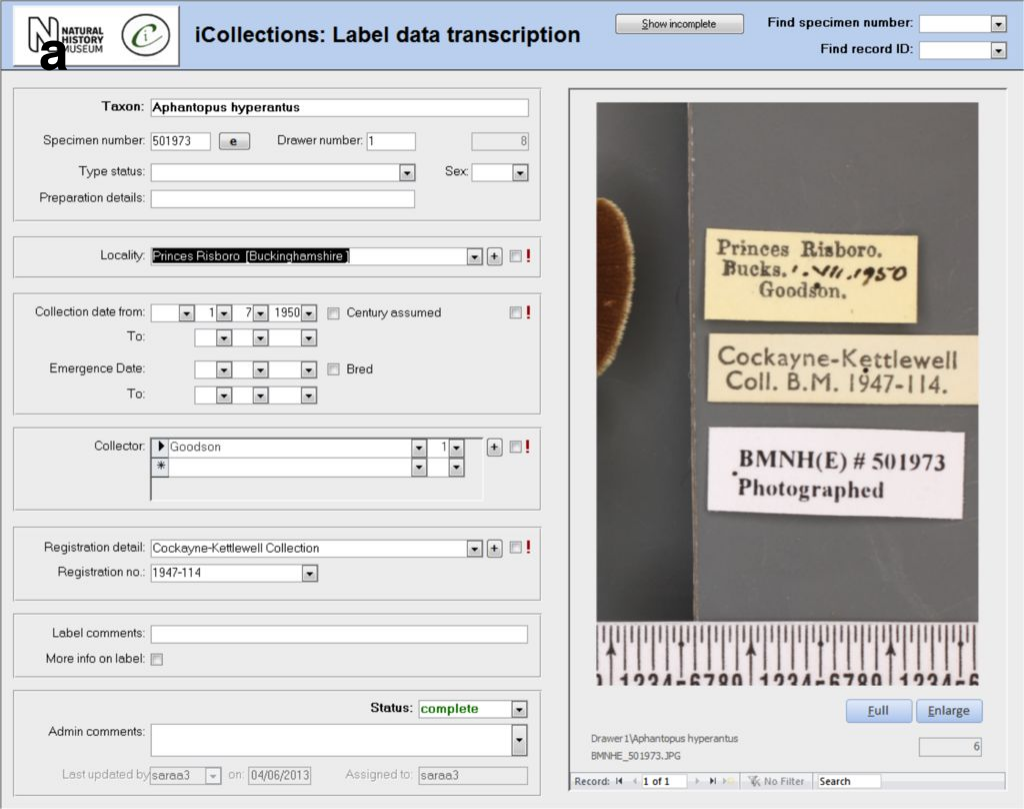
Transcription

**Figure 4b.**
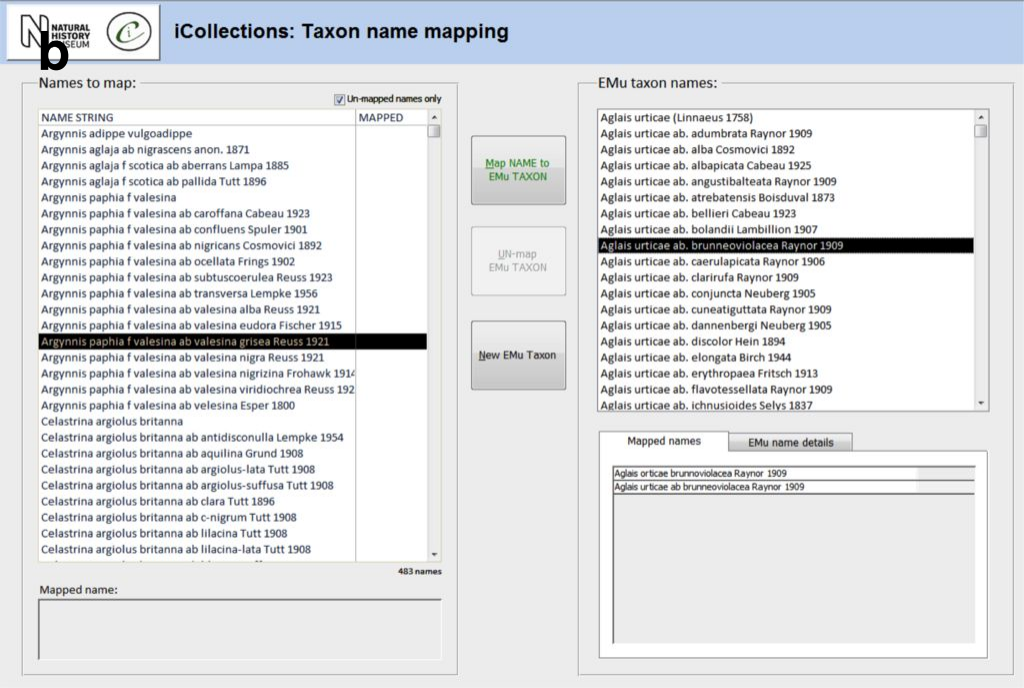
Taxon normalisation

**Figure 4c.**
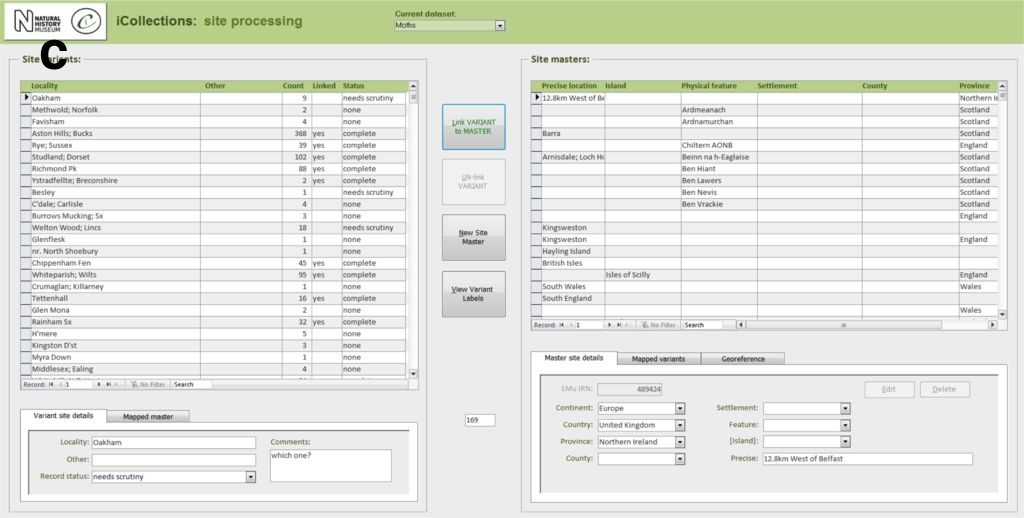
Site normalisation

**Figure 4d.**
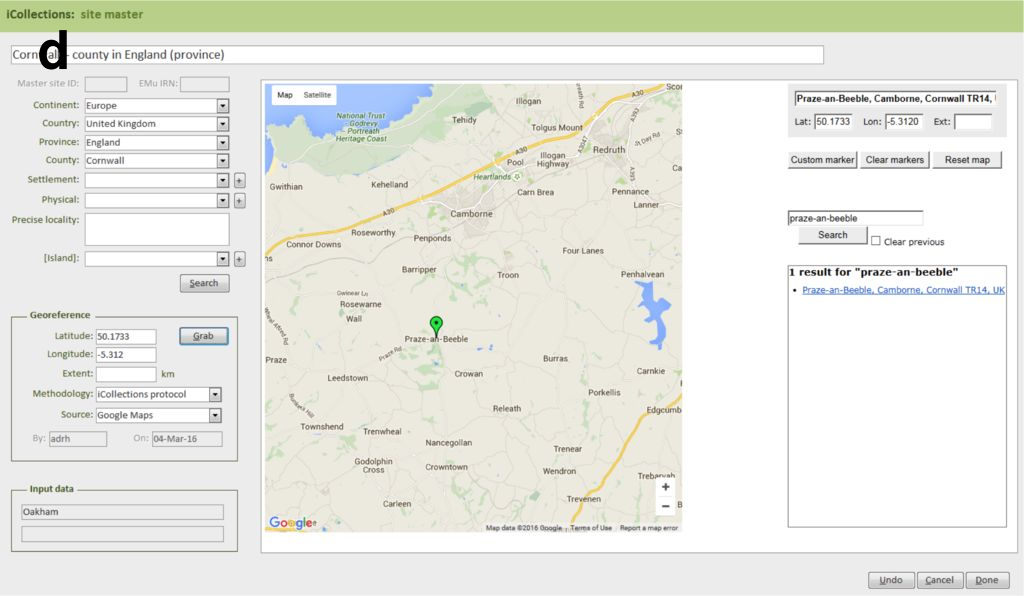
Georeferencing

**Figure 8a.**
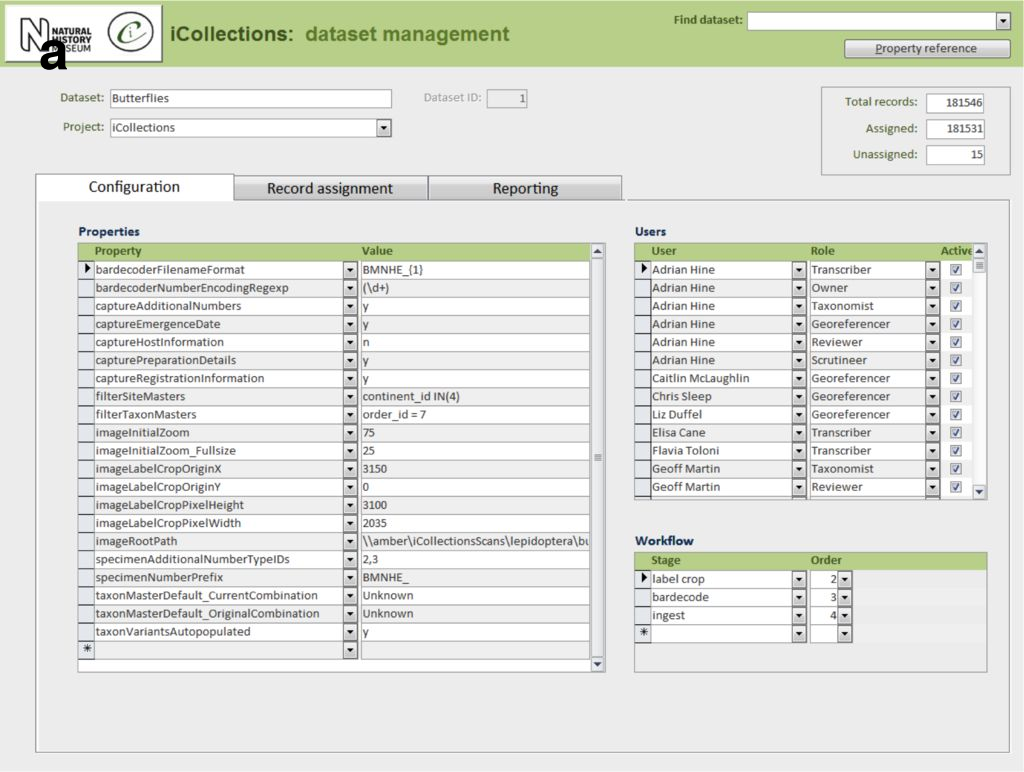
Configuration

**Figure 8b.**
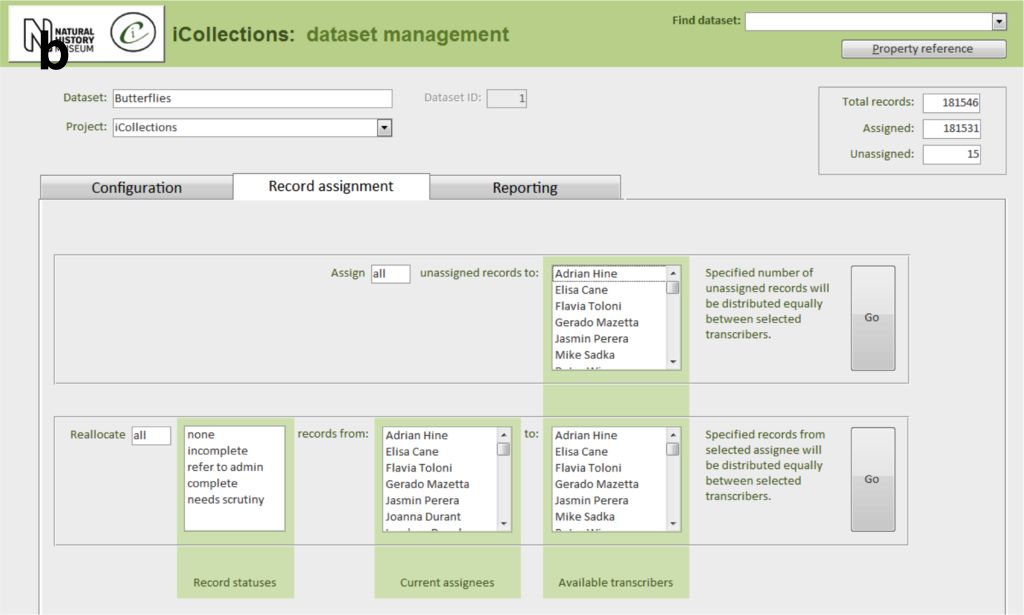
Record assignment

**Figure 8c.**
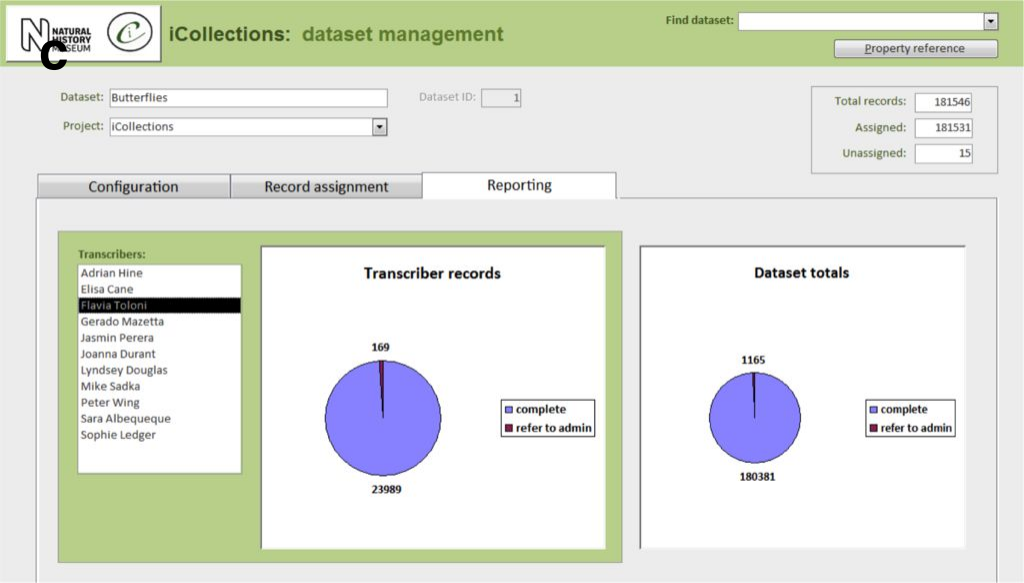
Reporting

**Table 1.** Number of taxa, specimens and images in different projects.

**Collection**	**Number of taxa**	**Number of specimens**	**Number of images**	**Total size, Gb (estimated)**
Butterflies	100	181545	184628	1133
Moths	331	199141	224459	1377
CWR Diptera	2478	33365	33365	165
CWR (excluding Diptera)	1138	24520	24520	121

**Table 2.** Georeferencing statistics for different projects.

**Project**	**Site Variants**	**Specimens**	**Site Masters**	% **reduction**	**Top 10 sites represent, specimens**	**Top 100 sites represent, specimens**	**Median no. of specimens/site**	**Mean no. of specimens/site**
Butterflies	9,591	183,000	4835	50%	52973	111589	4	43.7
Moths	~8000	1,000,000	~5000	?	?	?	?	?
CWR:Psyllids	1,876	12,500	1236	35%	1073	4112	3	7.1
CWR:Beetles and Diptera	10,353	50,000	5429	48%	3223	13394	2	9

**Table 3.** Data available after transcription.

**Data from iCollections Butterflies**	**Site**	**Date**	**Collector**	**No. of Records**	**Percentage of Total**
Site + Date + Collector	X	X	X	100,798	56%
Site + Date only	X	X		39,869	22%
Site + Collector only	X		X	16,942	9%
Site only	X			8,966	5%
Collector only			X	3,968	2%
Date only		X		771	0.4%
Date + Collector only		X	X	688	0%
None				9,543	5%
Total records				181,545	100%

**Table 4.** Data transcribed from collection labels.

**Research**	**Collections Management**	**Not Transcribed**
Taxonomic determination	Unique object identifier	Prior collections
Georeferenced locality	Drawer number	Sale information
Collection date	Type status	Unknown numbers
Breeding information	Registration details	Spurious information
	Collector(s)	
	Preparation details	

**Table 5.** Actions for different record types during import.

**Entity**	**Action in EMu**	**Comments**
Catalogue (specimens)	create new	Existing KE EMu specimen records from the Cockayne project (n=1691) were deleted and replaced by corresponding new records.
Multimedia (images)	create new	Up to two full images per specimen. Label crops added as separate records.
Locations	match to existing	A new set of 5000 drawers records was created to match against those pre-existing
Taxonomy	match to existing or create new	Most taxon records were imported into iCollections from EMu, so were already matched to existing EMu records. New taxa were matched or created as appropriate.
Parties (collectors)	match to existing or create new	Undertaken only for simple data easily matched to KE EMu parties concepts. Transcribed collector data was not augmented or interpreted, but there can be further enhancement later if warranted.
Collection Events	create new	The likelihood of matching existing KE EMu collection event records is small so these were created new.
Sites	create new	Existing site records in KE EMu records are not of sufficient quality and lack georeferences. All master sites from iCollections were created as new site records in EMu.

**Table 6.** Performance of digitisers in different projects, minutes per specimen.

Butterflies				
	Preparation	Imaging	Transcription	TOTAL
MIN	0.13	0.47	0.05	0.52
MAX	1.50	1.88	1.14	4.52
MEDIAN	0.85	0.93	0.53	2.32
BASE	0.84	1.01	0.55	**2.40**
SD	0.23	0.23	0.18	0.67
%	35%	42%	23%	
Moths				
	Preparation	Imaging	Transcription	TOTAL
MIN	0.42	0.48	0.30	1.20
MAX	1.81	1.96	1.64	5.40
MEDIAN	1.00	1.12	0.90	3.02
BASE	1.04	1.16	0.92	**3.11**
SD	0.23	0.25	0.22	0.70
%	33%	37%	30%	
Diptera				
	Preparation	Imaging	Transcription	TOTAL
MIN	0.66	0.74	0.58	1.99
MAX	1.88	2.36	2.67	6.90
MEDIAN	1.06	1.25	1.17	3.48
BASE	1.13	1.35	1.32	**3.80**
SD	0.20	0.27	0.35	0.82
%	30%	36%	35%	

**Table 7.** Project costs and duration (time is shown without georeferencing).

	Butterflies	Moths	CWR
Expenses			
Digitiser salaries	£171,465.40	£200,949.57	£82,776.40
Georeferencing	£19,907.72	£20,000.00 (est.)	£21,489.38
Project staff salaries	£158,198.58	£70,945.91	£28,522.03
Office preparation	£6,181.80	£0.00	£0.00
Equipment	£14,718.38	£0.00	£0.00
Total	£**370,471.88**	£**291,895.48**	£**132,787.81**
Time, person*months	87	101	42
Cost per specimen			
Total	£2.02	£1.47 (est.)	£2.29
Without project staff (digitisers only)	£1.17	£1.11	£1.80
Georeferencing	£0.11	£0.10 (est.)	£0.37
